# Retinal Vein Passing through a Congenital Optic Nerve Pit

**DOI:** 10.18502/jovr.v16i2.9101

**Published:** 2021-04-29

**Authors:** Hasan Kiziltoprak, Kemal Tekin, Alper Dilli, Mehmet Yasin Teke

**Affiliations:** ^1^Ankara Ulucanlar Eye Training and Research Hospital, Ankara, Turkey; ^2^Ercis State Hospital, Van, Turkey; ^3^Diskapi Yildirim Bayazit Education and Research Hospital, Radiology Department, Ankara, Turkey; ^4^Department of Retinal Diseases, Ankara Ulucanlar Eye Training and Research Hospital, Ankara, Turkey

##  PRESENTATION

We describe a 65-year-old woman with a retinal vein exiting via the pit of the optic nerve (PON). The patient did not report any history of ocular or systemic problems.

**Figure 1:**
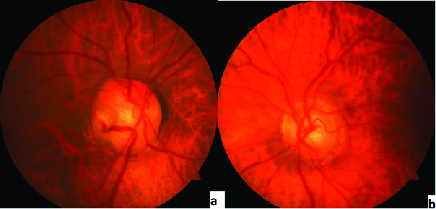
(a&b) Fundus photograph of the patient demonstrates a temporal pit of the optic nerve (PON) and a retinochoroidal vein that sinks into the PON in the right eye and a normal optic disc in the left eye.

**Figure 2:**
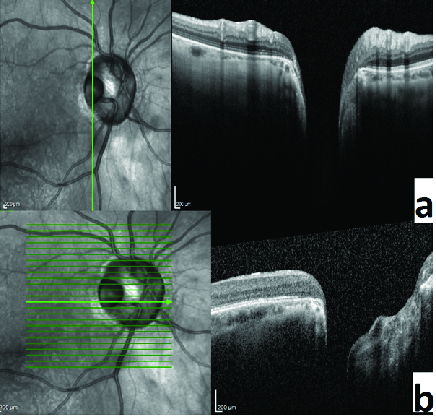
(a&b) Horizontal and vertical sections of the spectral domain optical coherence tomography scans of the right eye show the presence of cavitation in the disc stroma.

**Figure 3:**
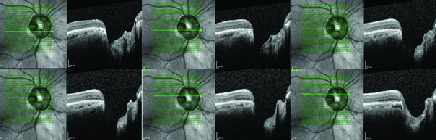
Horizontal sections of the spectral domain optical coherence tomography scans show the relationship of the pit of the optic nerve (PON) with the retinochoroidal vein.

**Figure 4:**
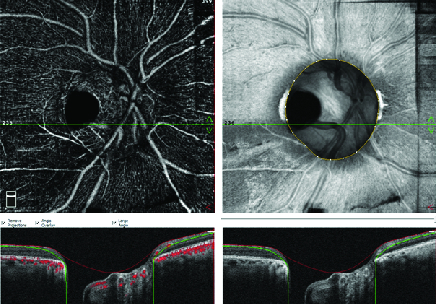
Optical coherence tomography angiography demonstrates cavitation in the disc stroma and a retinochoroidal vein that sinks into the pit of the optic nerve (PON) at the level of the superficial capillary plexus in the right eye.

**Figure 5:**
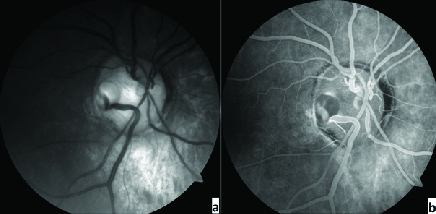
(a&b) Fundus fluorescein angiography shows the filling of the retinochoroidal vein in the early and late phases.

A temporal congenital pit of the optic nerve (CPON) was detected in the right eye of a 65-year-old woman on routine examination. A retinal vein was noted to exit directly through the optic pit. The vein possibly continued as a choroidal vessel visible in continuity with the retinal vein [Figure 1a]. The left optic nerve was normal [Figure 1b]. Horizontal and vertical sections of the spectral domain optical coherence tomography (SD-OCT) scans of the right eye showed cavitation in the disc stroma [Figures 2a and 2b]. Horizontal sections of the SD-OCT scans showed the relationship of the PON with the retinochoroidal vein [Figure 3]. OCT angiography demonstrated cavitation in the disc stroma and a retinochoroidal vein that emerged through the PON at the level of the superficial capillary plexus in the right eye [Figure 4]. Fundus fluorescein angiography showed filling of the retinochoroidal vein in the early and late phases [Figures 5a and 5b]. Magnetic resonance imaging with contrast of the brain and optic disc showed no venous malformation or vascular anastomosis.

##  DISCUSSION

Pit of the optic nerves can be congenital or acquired and consists of the presence of a crater-like depression in the optic nerve head.^[1,2]^ CPONs are typically temporal.^[1]^ Acquired disc pits occur in association with myopia and glaucoma.^[1]^ CPON is usually asymptomatic and diagnosed incidentally; however, serous retinal detachment (SRD) or cystoid retinal edema are potential complications.^[2,3,4]^

To the best of our knowledge, this is the first report of a retinal vein exiting the eye via an optic pit. We propose that the vein is retinochoroidal as it seems to continue as a choroidal vein. That retinal vein may also be a retina–pial vein connecting the retinal circulation to the pial vessels of the optic nerve dura through the pit. However, there is no clear evidence of an anastomosis between the pial or posterior ciliary vessels of the optic nerve with the retinal vein.

##  Informed consent

Informed consent was obtained from the patient included in the study.

##  Financial Support and Sponsorship

Nil.

##  Conflicts of Interest

The authors declare that there is no conflict of interest regarding the publication of this paper.
